# Activation of AMPK Entails the Protective Effect of Royal Jelly against High-Fat-Diet-Induced Hyperglycemia, Hyperlipidemia, and Non-Alcoholic Fatty Liver Disease in Rats

**DOI:** 10.3390/nu15061471

**Published:** 2023-03-18

**Authors:** Alaa Hasanain Felemban, Ghedeir M. Alshammari, Abu ElGasim Ahmed Yagoub, Laila Naif Al-Harbi, Maha H. Alhussain, Mohammed Abdo Yahya

**Affiliations:** Department of Food Science and Nutrition, College of Food and Agricultural Science, King Saud University, Riyadh 11451, Saudi Arabia

**Keywords:** royal jelly, NAFLD, AMPK, hyperglycemia, hyperlipidemia

## Abstract

This study examined the mechanism underlying the protective effect of royal jelly (RJ) against high-fat-diet (HFD)-mediated non-alcoholic liver disease (NAFLD) in rats. Adult male rats were divided into five groups (n = 8 each): control fed a standard diet, control + RJ (300 mg/kg), HFD, HFD + RJ (300 mg/kg), and HFD + RJ + CC (0.2 mg/kg). The treatment with RJ reduced weight gain, increased fat pads, and attenuated fasting hyperglycemia, hyperinsulinemia, and glucose tolerance in the HFD-fed rats. It also reduced the serum levels of liver function enzymes, interleukin 6 (IL-6), tumor necrosis factor-α (TNF-α), and leptin but significantly increased the serum levels of adiponectin. In addition, and with no effect on lipid excretion in stool, RJ significantly decreased the hepatic mRNA expression of SREBP1, serum, hepatic cholesterol, and triglycerides but increased hepatic mRNA levels of PPARα. Furthermore, RJ reduced the hepatic levels of TNF-α, IL-6, and malondialdehyde (MDA) in the livers of these rats. Of note, with no effect on the mRNA levels of AMPK, RJ stimulated the phosphorylation of AMPK and increased the levels of superoxide dismutase (SOD) and total glutathione (GSH) in the livers of the control and HFD-fed rats. In conclusion, RJ attenuates NAFLD via its antioxidant potential and adiponectin-independent activation of liver AMPK.

## 1. Introduction

Non-alcoholic fatty liver disease (NAFLD) is the most common liver disorder associated with obesity, metabolic disorder, and insulin resistance (IR) [1]. The disease is characterized by the exaggerated synthesis and accumulation of triglycerides (TGs) and cholesterol (CHOL) in the liver (steatosis), which can then be progressively converted to more severe conditions such as non-alcoholic steatohepatitis (NASH), fibrosis, and cancer [1]. By 2030, NAFLD will be considered the most frequent cause of liver transplantation [2].

The pathogenesis of NAFLD is currently well-established [3]. It is widely accepted that peripheral IR, mainly due to stimulated lipolysis in the insulin-unresponsive adipose tissue, is the major cause underlying NAFLD [4]. In this regard, it was shown that IR leads to a massive increase in inflammatory cytokines and free fatty acids (FFAs) from the adipose tissue to the liver, which can then promote a state of inflammation and oxidative stress by activating resident inflammatory cells, mitochondria damage, and endoplasmic reticulum (ER) stress [5]. In addition, hepatic oxidative stress and inflammation can worsen the condition and result in sustained hyperglycemia by promoting hepatic IR [6]. Indeed, animals and patients with NAFLD exhibit the increased generation of reactive oxygen species (ROS) and high concentrations of inflammatory cytokines in their serum and livers [7,8]. However, suppressing adiposeness by stimulating peripheral insulin action, as well as treatment with anti-inflammatory and antioxidant drugs, has shown very promising results in alleviating NAFLD and its complications in both humans and animals [9].

During the last decades, several trials have been conducted to identify suitable safe drugs that prevent or treat NAFLD. To date, calorie restriction and exercise are the only available and approved options to treat NAFLD [10,11]. However, more research is now focusing on identifying safe pharmaceutical drugs that can manipulate the important key signaling pathways and mechanisms involved in the pathogenesis of NAFLD, such as de novo lipogenesis and mitochondria biogenesis [11]. One interesting target is the highly conserved heterotrimeric serine/threonine protein kinase complex known as 5′AMP-activated protein kinase (AMPK) [12]. AMPK depends on the liver, muscles, and adipose tissue and is activated during starvation with increasing ratios of adenosine monophosphate (AMP)/adenosine triphosphate (ATP). The ability of AMPK to treat NAFLD has been established in numerous animal and preclinical studies. It has been shown that the levels of AMPK activation are substantially decreased in animals and patients with NAFLD, whereas the activation of AMPK by genetic manipulation, exercise, activators (e.g., A-769662, EX229/991, MT47-100), and mitochondria inhibitors (e.g., metformin) showed protective effects [12,13,14,15,16,17]. Researchers have demonstrated that the protection afforded by AMPK is related to its ability to act using different mechanisms, including improving peripheral insulin sensitivity; suppressing de novo lipogenesis and lipogenic genes (e.g., sterol regulatory element-binding protein 1 (SREBP-1c) and acetyl-CoA carboxylase (ACC-1)); and stimulating adipose tissue, liver fatty acid oxidation, and mitochondria integrity/function [13].

Royal jelly (RJ) is one of the most nutritional foods available globally. It is produced by the mandibular glands of honeybees and is the main food for their queen [18]. The major constituents of RJ are water, protein, carbohydrates, lipids, polyphenols, and vitamins [19]. RJ is hypotensive and has potent antidiabetic activity due to its ability to stimulate insulin release, improve insulin sensitivity, and control glycemia [18,20,21]. In addition, RJ has been shown to alleviate neural, renal, and hepatic damage in several conditions, and it has potent antibacterial and antiaging effects due to its well-known antioxidant and anti-inflammatory properties [18]. Furthermore, RJ has been shown to attenuate dyslipidemia and to reduce circulatory levels of TGs, cholesterol (CHOL), and low-density lipoproteins (LDL-c) in obese and diabetic individuals, as well as in several experimental animal models [22,23,24,25,26,27]. However, the precise mechanism underlying RJ’s hypoglycemic and hypolipidemic effects is still unknown. In their most recent article, Malekia et al. [18] recommended looking at the effect of RJ on AMPK signaling.

Therefore, in this study and using control and high-fat-diet-fed rats, we aimed to examine the effect of RJ on the major genes involved in lipogenesis and FA oxidation such as SREBP1 and the peroxisome proliferator-activated receptor alpha (PPARα). In addition, we tested the hypothesis that the hypolipidemic effect of RJ in these NAFLD animals is mediated mainly by regulating AMPK.

## 2. Materials and Methods

### 2.1. Animals

Fifty-six adult male Wistar albino rats were supplied from the Experimental Animal Care Center at King Saud University (KSU), Riyadh, Saudi Arabia. All animals were housed under controlled ambient conditions (22 ± 2 °C and 55 ± 5% relative humidity) and exposed to a daily light/night cycle of 12 h each. During the one-week adaptation period and throughout the experimental procedures, all rats had free access to their food and drinking water. All experimental procedures conducted in this study were approved by the Research Ethics Committee at KSU, Riyadh, Saudi Arabia, which follows the well-established guidelines of the National Research Council’s Guide for the Care and Use of Laboratory Animals [28].

### 2.2. Diets

Chronic feeding of an HFD is the most common protocol to induce NAFLD in rats [29]. Commercially available standard and high-fat diets (STD and HFD, respectively) were used in this study. The STD (Cat. No. Teklad 2014) was purchased from Envigo, Indianapolis, IN, USA, and has a total energy of 2.9%, of which 13% is obtained from fat. The HFD (Cat. No. D12451) was purchased from Research Diets, New Brunswick, NJ, USA, and has a total energy of 4.73 kcal/g, where 45% of this energy is obtained from fat. This diet was used previously to induce IR and NAFLD in rats [30].

### 2.3. Experimental Design

Fresh royal jelly (RJ) was purchased from a certified supplier in Riyadh, KSA, and was identified and its purity tested by an expert nutritionist at the department of nutrition at KSU. A total of 56 male rats were selected randomly and divided and classified as follows (n = 8 rats/group): (1) control group: fed STD for 16 weeks and received normal saline as a vehicle; (2) control + RJ (300 mg)-treated groups: fed STD for 16 weeks and co-treated with RJ at a final concentration of 300 mg/kg; (3) HFD model group: fed HFD for 16 weeks and co-treated with normal saline as a vehicle; (4) HFD + RJ (300 mg)-treated groups: fed an HFD for 16 weeks and co-treated with RJ at a final concentration of 150 mg/kg; (5) HFD + RJ (300 mg) + CC-treated rats: fed an HFD for 16 weeks and co-treated orally with RJ at a final concentration of 300 mg/kg and intraperitoneally (i.p.) with dorsomorphin (compound C/CC) (0.2 mg/kg). Normal saline and RJ were always given by gavage at 7:00 a.m. Body weight, food intake, and calorie intake were monitored every 2 days. The in vivo dose of CC was based on previous studies [30]. The dose of RJ was based on the study of several authors who have shown the ability of RJ to reduce fasting glucose, TGs, CHOL, and LDL-c improve insulin action in diabetic and hyperlipidemic rats [27,31,32,33].

### 2.4. Oral Glucose Tolerance Test (OGTT)

The oral glucose tolerance test (OGTT) was performed as described during the last 3 days of the experimental procedure. Briefly, blood samples were drawn from overnight fasted rats, and then all rats from all groups were orally administered glucose (2 g/kg). Blood samples were redrawn at different time intervals over 2 h after glucose administration in EDTA-containing tubes, centrifuged (1100× *g*/10 min), and then the plasma was collected. These samples were used to measure the levels of glucose and insulin using rat-specific ELISA kits (Cat. No. 10009582, Cayman Chemicals, Ann Arbor, MI, USA, and Cat. No. 589501, Ann Arbor, TX, USA, respectively). As a measure of IR, the homeostasis model assessment of IR (HOMA) was calculated from fasting glucose and insulin levels using the formula described by Yoon et al. [34]: HOMA-IR = ((glucose [mg/dl] × insulin (ng/mL))/405). All measurements were duplicated for eight samples/groups according to the manufacturer’s instructions for each kit. 

### 2.5. Blood and Tissue Sampling

By the end of the experimental procedure, all animals were fasted overnight and were anesthetized (ketamine/xylazine; 80:10 mg/mg). Blood samples (1 mL) were collected by cardiac puncture, centrifuged (1100× *g*/10 min), and the serum was collected. This serum was stored at −80 °C and used later for biochemical analysis. Then, all rats of all groups were killed by cervical dislocation, and their liver and white adipose tissue (inguinal, epididymal, peritoneal, and mesenteric) were collected on ice and weighed. Parts of the livers were fixed in 10% buffered formalin for histological evaluation, and all remaining parts, as well as WAT pads, were snap-frozen in liquid nitrogen and stored at −80 °C until further use. The stools of each group of rats were collected during the last 2 weeks of the experiment, pooled, dried at 37 °C, and stored at 4 °C.

### 2.6. Hepatic and Stool Lipid Extraction and Preparation of Tissue Homogenates

The chloroform–methanol described by Folch et al. [35] was used to extract various lipid fractions from the stools and livers. In brief, 0.25 g of the frozen liver tissues or collected stools were homogenized in a 10 mL methanol/chloroform mixture (1:2; *v*/*v*) and then incubated for 5 h. Normal saline (5 mL) was added to the mixture and then centrifuged at a rate of 2000× *g* for 20 min. This allows the separation of two layers. The lower layer was separated, and the solvent was evaporated by a rotatory evaporator where the solid lipids were redissolved in 1 mL of isopropanol, stored at 4 °C, and then used directly to measure the lipid levels. In addition, some parts of the frozen livers were homogenized in ice-cold phosphate-buffered saline (PBS/pH −7.4) and centrifuged at 1200× *g* for 15 min to collect supernatants. These supernatants were stored at −20 °C and used later for biochemical analysis in tissue homogenates.

### 2.7. Biochemical Analysis

Serum, hepatic, and stool levels of TGs and CHOL were measured using commercial assay kits (Cat. No. ECCH-100, BioAssay Systems, Hayward, CA, USA and Cat. No. 10009582, Cayman Chemicals, Ann Arbor, MI, USA). The hepatic and serum levels of FFAs were measured using colorimetric kits (Cat. No. MBS014345, MyBioSource, San Diego, CA, USA). The serum levels of high-density lipoprotein–cholesterol (HDL-c) and low-density lipoprotein–cholesterol (LDL-c) were measured using the following assay kits (Cat. No. STA-394, Cell Biolabs, San Diego, CA, USA; Cat. No. 79960; Crystal Chemicals, Houston, TX, USA). The serum levels of aspartate aminotransferase (AST), alanine aminotransferase (ALT), and gamma-glutamyl transpeptidase (GGT) were measured using rat-specific ELISA kits (Cat. No. MBS264975; Cat. No. MBS269614; Cat. No. MBS9343646, MyBioSource, San Diego, CA, USA, respectively). The serum levels of leptin and adiponectin were measured using rat-specific ELISA kits (Cat. No. ab100773 and Cat. No. ab239421, Abcam, Cambridge, UK). The hepatic levels of total glutathione (GSH), malondialdehyde (MDA), and superoxide dismutase (SOD) were measured using the following ELISA kits: Cat. No. MBS265966, Cat. No. MBS268427, and Cat. No. MBS036924, MyBioSource, San Diego, CA, USA, respectively. Finally, the hepatic levels of tumor necrosis factor-alpha (TNF-α) and interleukin 6 (IL-6) were measured by ELISA (Cat. No. BMS622, Thermo Fisher, Bremen, Germany; and Cat. No. R6000B R&D System, Minneapolis, MN, USA, respectively). All measurements were conducted for eight samples/groups as per the kits’ instructions.

### 2.8. Real-Time PCR

The mRNA levels of markers of AMPKα, SREBP-1c, PPARα, and GAPDH (the reference gene) were measured in the liver of each rat. The primer pair sequences have been previously validated in our laboratories and described by us and other authors [30,36]. In brief, the total RNA was extracted using a commercial kit (Cat. No. 74004; Qiagen, Hilden, Germany). The purity of the RNA was determined using the absorbance 260/280. The first-strand cDNA was synthesized using the supplied commercial kit (Cat. No. K1621, Thermo Fisher kit). The amplification of the mRNA was conducted using the SsoFast EvaGreen Supermix kit (Cat. No. 172-5200, Bio-Rad, Hercules, CA, USA) and Bio-Rad qPCR amplification (model CFX96) as instructed by the kit. The following steps were followed for each target: (1) heating (1 cycle/98 °C/30 s), (2) denaturation (40 cycles/98 °C/5 s), (3) annealing (40 cycles/60 °C/5 s), and (4) melting (1 cycle/95 °C/5 s/step). The relative mRNA expression of all target genes was presented after the normalization of GAPDH using the 2ΔΔCT method.

### 2.9. Western Blotting

For Western blotting, the liver tissues were homogenized in radioimmunoassay (RIPA) buffer (Cat. No. 89901, Thermo Fisher, Waltham, MA, USA). The total protein levels in all samples were measured using the Pierce™ BCA Protein Assay Kit (Cat. No. 23225, Thermo Fisher, Waltham, MA, USA). The proteins were diluted in the loading buffer, and then equal volumes of each sample were separated by the SDS-PAGE. The proteins were then transferred to nitrocellulose membranes, blocked with 5% skimmed milk, and incubated with primary antibodies against total and phospho-AMPK (Cat. No. 2532 and Cat. No. 2531; Cell Signaling Technology, Danvers, MA, USA; 62 kDa, 1:1000 and 1:500, respectively) or β-actin (# 3700, 45 kD, 1:1000). The membranes were then incubated with the corresponding secondary antibodies and incubated with West Pico PLUS chemiluminescence substrate (Cat. No. 34580, Thermo Fisher, Waltham, MA, USA) for 5 min. The developed bands were scanned and analyzed using the C-Di Git blot scanner. Washing three times for 10 min with TBST buffer was conducted between steps. All antibodies, as well as the skimmed milk, were diluted in the TBST buffer. Incubations with the primary or secondary antibodies were performed at room temperature for 2 h and with continuous shaking. The expression of all target proteins was normalized against β-actin.

### 2.10. Liver Histopathological Evaluation

Cuts of liver samples were immersed in 10% buffered formalin for 24 h. All tissues were dehydrated in increasing concentrations of ethanol and were then cleaned with xylene. The tissues were subsequently coated in paraffin wax and sectioned with a microtome (5 µm). The tissues were then routinely stained with hematoxylin and eosin (HE) for overall morphology. All photos were captured using a light microscope at a magnification of 200×.

### 2.11. Statistical Analysis

All data were fed into a computer and analyzed by two-way ANOVA using GraphPad Prism software. Normality was tested using the Kolmogorov–Smirnov test. The comparison between the various groups was conducted using Tukey’s post hoc test. The data were considered significantly different at *p* < 0.05.

## 3. Results

### 3.1. RJ Reduces Body and WAT Fat Weights with No Effect on Food Intake

**Figure 1:**
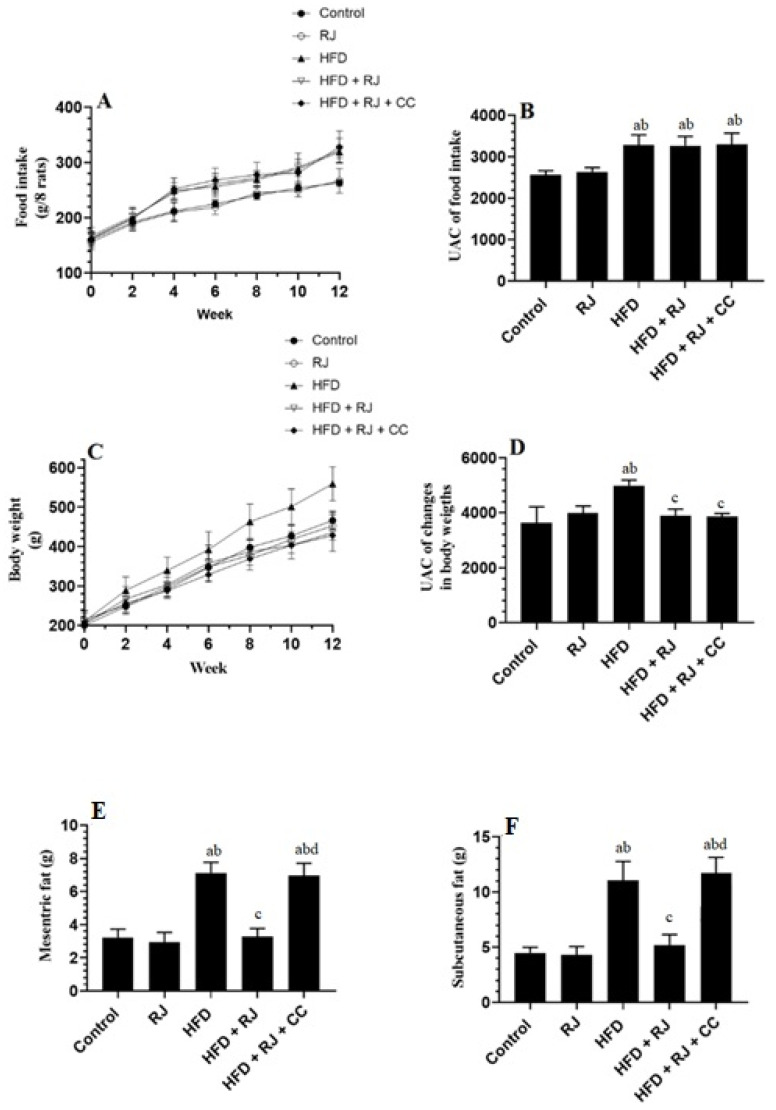
Changes in food intake and its corresponding area under the curve (**A**,**B**), changes in body weights and its corresponding UAC (**C**,**D**), and the weights of mesenteric and subcutaneous fats (**E**,**F**) in all experimental groups. Data are presented as means ± SD (n = 8/group). ^a^: vs. control; ^b^: vs. RJ-treated control rats; ^c^: vs. HFD-model rats; ^d^: vs. HFD + RJ-treated rats. RJ: royal jelly; HFD: high-fat diet; CC: compound C, an AMPK inhibitor.

Final body weights, weekly food intake, and weights of the mesenteric and subcutaneous fats were significantly increased in the HFD-fed rats compared to the control rats (Figure 1A–E). However, treatment with RJ with or without CC did not alter the food intake in the HFD-fed rats (Figure 1A,B). In the same way, treatment with RJ had no effect on the food intake in the control rats (Figure 1A,B). In contrast, although no effect was shown in the control rats, RJ reduced the final body weights and the weights of subcutaneous and mesenteric fats in the HFD-treated rats. These effects were prevented when CC was co-administered with RJ (Figure 1B–F).

### 3.2. RJ Attenuates Fasting Hyperglycemia, Insulinemia, and Insulin Resistance in HFD-Fed Rats

**Figure 2:**
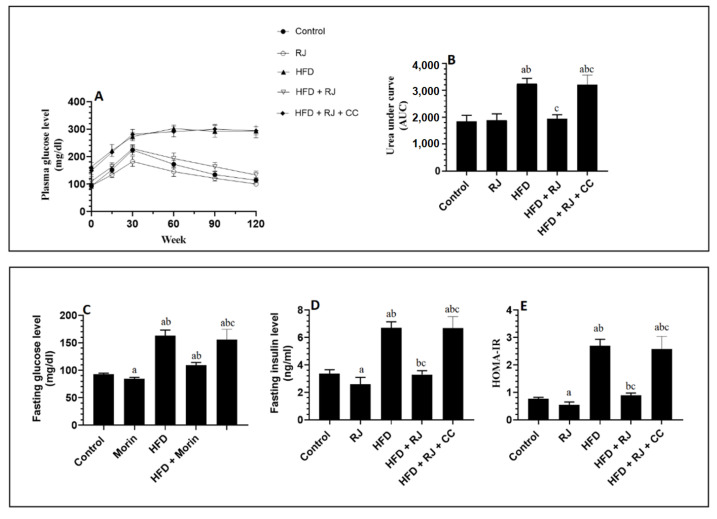
Changes in glucose levels during the oral glucose tolerance test (OGTT) and its corresponding area under the curve (AUC) (**A**,**B**), as well as changes in the fasting plasma glucose (**C**), fasting plasma insulin (**D**), and HOMA-IR (**E**) in all groups of rats. Data are presented as means ± SD (n = 8/group). ^a^: vs. control; ^b^: vs. RJ-treated control rats; ^c^: vs. HFD-model rats; RJ: royal jelly; HFD: high-fat diet; CC: compound C, an AMPK inhibitor.

The serum glucose levels, as presented by the graph or area under the curve (AUC), were significantly increased from 0.0 min until the end of 120 min of the OGTT in the HFD-fed rats compared to the control and RJ-treated control rats (Figure 2A,B). The AUC for the glucose levels of the OGTT measured in the HFD + RJ-treated rats were not significantly different from the control or RJ-treated rats (Figure 2A,B). No significant variations in the glucose levels presented on the 120 min graph or calculated AUC were seen between the HFD-model rats and HFD + RJ + CC-treated rats. In accordance, the fasting glucose, insulin, and HOMA-IR index were significantly higher in the HFD-fed rats than in the control or RJ-treated rats but were significantly reduced in the HFD + RJ-treated rats (Figure 2C–E). On the other hand, no significant variations in the levels of these biochemical markers were seen when the HF-fed rats were compared to the HFD + RJ + CC-treated rats (Figure 2C–E). Of note, the serum levels of fasting glucose, insulin, and HOMA-IR were significantly decreased in the RJ-treated control rats compared to the control rats that received the vehicle (Figure 2C–E).

### 3.3. RJ Improves Adiponectin Levels and Ameliorates Systemic Inflammation and the Increase in Liver Marker Enzymes in HFD-Fed Rats

The liver weights and the serum levels of leptin, TNF-α, IL-6, ALT, AST, and γ-GTT were significantly increased, but the serum levels of adiponectin were significantly decreased in the HFD-fed rats compared to the control rats and were significantly reversed in the HFD + RJ-treated rats (Table 1). Among these markers, only the serum levels of adiponectin were significantly increased in the RJ-treated control rats compared to the control rats administered the vehicle (Table 1). However, the liver weights and the levels of all these markers did not significantly vary between the HFD and HFD + RJ + CC-treated rats (Table 1).

### 3.4. RJ Reverses Hyperlipidemia and Reduces Hepatic Lipid Levels in HFD-Fed Rats

The HFD-fed rats showed a significant increment in the serum and hepatic levels of CHOL, FFAs, and TGs compared to the control rats (Table 2). Their serum had higher levels of LDL-c, and their stool showed significantly higher levels of CHOL and TGs (Table 2). The fecal levels of CHOL and TGs were not significantly different when the control rats were compared with the RJ-treated control rats or when the HFD-fed, HF + RJ, and HFD + RJ + CC rats were compared with each other (Table 2). On the other hand, the RJ-treated control rats showed significantly lower serum and hepatic levels of TGS, FFAs, and CHOL as well as lower levels of LDL-c compared to the control rats (Table 2). In addition, a similar reduction in the levels of all these serum and hepatic lipid markers was seen in the HFD + RJ-treated rats compared to the HFD-fed rats (Table 2). Interestingly, no significant differences in the levels of these hepatic and serum lipids were seen between HFD-fed rats and HFD + RJ + CC-treated rats (Table 2).

### 3.5. RJ Attenuates Oxidative Stress and Inflammation and Improves Antioxidant Status in the Livers of HFD-Fed Rats

**Figure 3:**
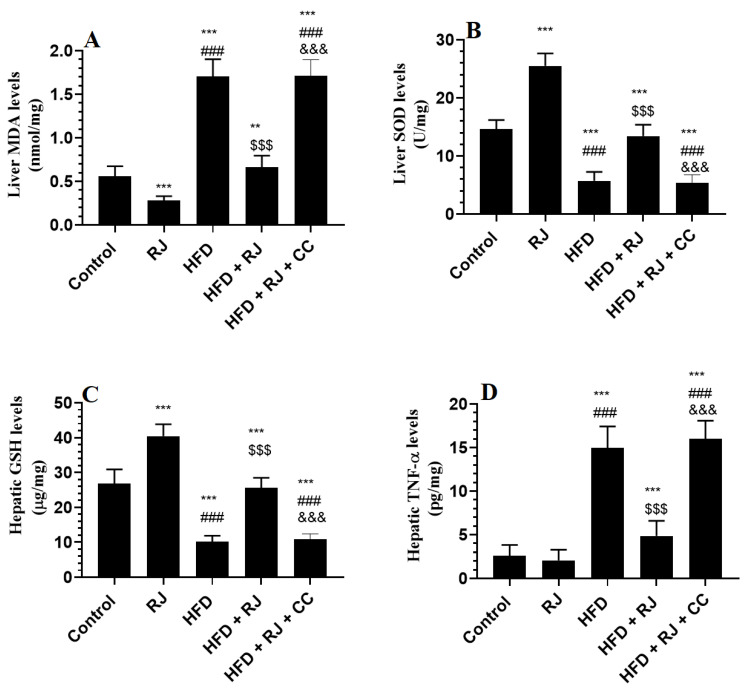
Markers of oxidative stress and inflammation in the livers of all experimental groups. Data are presented as means ± SD (n = 8/group). (**, ***) vs. control at *p* < 0.01 & 0.001, respectively; (**###**) vs. RJ-treated control rats at *p* < 0.001; (**$$$**) vs. HFD-model rats at *p* < 0.001; (**&&&**) vs. HFD + RJ-treated rats at *p* < 0.001. RJ: royal jelly; HFD: high-fat diet; CC: compound C, an AMPK inhibitor.

The hepatic levels of TNF-α, IL-6, and lipid peroxides (MDA) were significantly higher in the levels of total GSH and SOD in the livers of the HFD-fed rats compared to the control or RJ-treated rats (Figure 3A–D). The levels of GSH and SOD were significantly increased but the levels of TNF-α, IL-6, and MDA were significantly reduced in the livers of HFD + RJ-treated rats compared to the HFD-fed rats (Figure 3A–E). In addition, the levels of SOD and GSH were significantly higher but the levels of MDA were significantly lower in the livers of the RJ-treated control rats compared to the control rats administered the vehicle (Figure 3A–E). However, the levels of all these inflammatory/oxidant/antioxidant markers did not vary between the HFD + RJ + CC-treated rats and HFD-model rats (Figure 3A–E).

### 3.6. RJ Enhances the Activity (Phosphorylation) of AMPK and PPARα and Downregulates SREBP1c in the Livers of Control and HFD-Fed Rats

**Figure 4:**
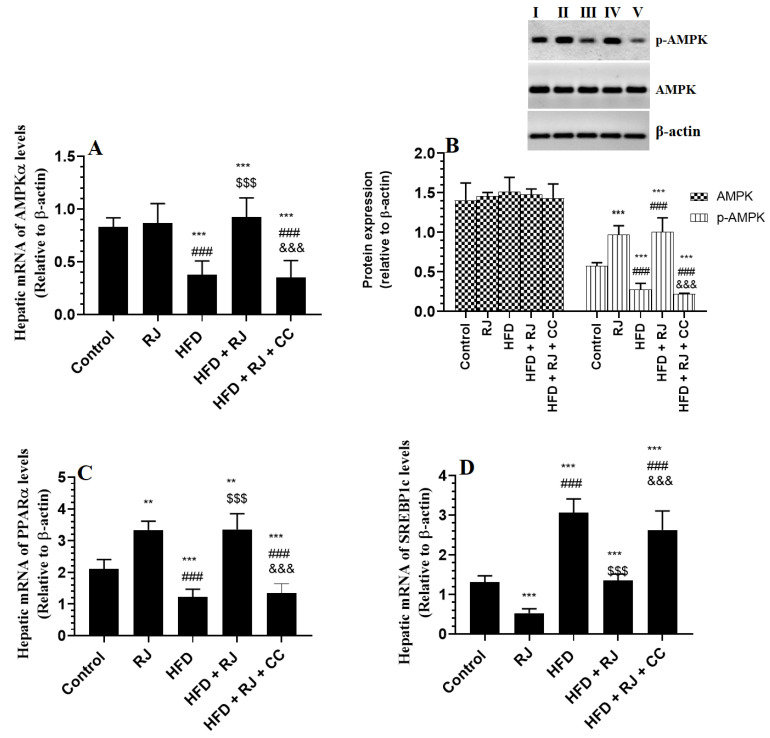
mRNA levels of AMPKα (**A**), total and phosphorylated protein levels of AMPKα (**B**), and mRNA levels of SREBP and PPARα (**C,D**) in the livers of all groups of rats. Data are presented as means ± SD (n = 8/group). (**, ***) vs. control at *p* < 0.01 & 0.001, respectively; (**###**) vs. RJ-treated control rats at *p* < 0.001; (**$$$**) vs. HFD-model rats at *p* < 0.001; (**&&&**) vs. HFD + RJ-treated rats at *p* < 0.001. RJ: royal jelly; HFD: high-fat diet; CC: compound C, an AMPK inhibitor. In the western blot of Figure **B**: lane I: control; lane B: RJ-treated rats, lane III: HFD; lane IV: HFD + RJ; lane V: HFD + RJ + CC-treated rats.

The mRNA levels and levels of p-AMPKα were significantly increased but the mRNA levels of SREBP1 and PPARα were significantly reduced in the livers of HFD-fed rats compared to the control and RJ-treated rats (Figure 4A–D). The total protein levels of AMPKα were not significantly different among all groups of the study (Figure 4D). The hepatic mRNA levels of AMPKα were not significantly different between the control and RJ-treated rats (Figure 4A). The hepatic mRNA levels of PPARα and SREBP1 were significantly reduced but the protein levels of p-AMPKα were significantly increased in the RJ-treated rats compared to the control rats (Figure 4A–D). This picture was reversed in the livers of HFD + RJ-treated rats compared to the HFD-fed rats. However, CC treatment did not alter the mRNA levels of AMPKα in the HFD + RJ + CC treated rats, but it significantly reduced the phosphorylation of AMPK and mRNA levels of PPARα and concomitantly increased the mRNA levels of SREBP1c (Figure 4A–D).

### 3.7. Histological Finding

**Figure 5:**
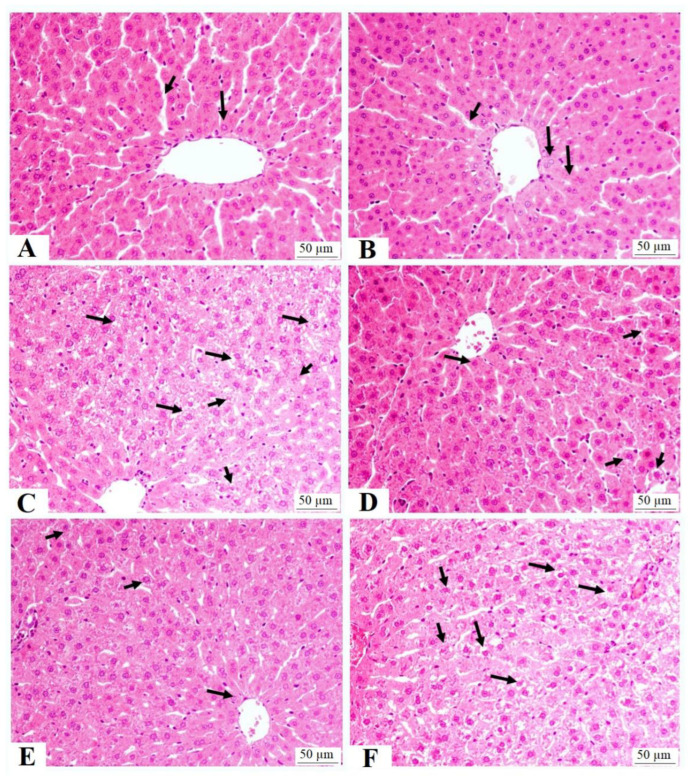
Liver histology in all groups of rats: (**A**,**B**) were taken from control and royal jelly (RJ)-treated rats and show normal features including central vein, hepatocytes (long arrow), and sinusoids; (**C**) was taken from high-fat-diet-fed rats and show an increased number of cytoplasmic vacuoles within the hepatocytes (long arrows) and necrotic cells (short arrows); (**D**,**E**) were taken from HFD + RJ-treated rats and show almost normal hepatocytes (long arrows) and sinusoids with few cytoplasmic vacuoles (short arrows); (**F**) was taken from HFD + RJ + compound C (CC)-treated rats and show a similar image to that seen in group C (HFD) with the maximum increase in the cytoplasmic vacuoles (long arrows) and necrotic cells (short arrows).

The livers of the control and RJ-treated rats showed normal features with intact central veins, sinusoids, and hepatocytes (Figure 5A,B). The livers from the HFD-fed rats showed an increased number of fat vacuoles of various sizes with dilated central veins. They also showed an increased number of necrotic cells (Figure 5C). Considerable improvement in the structure of the livers with very few fat vacuoles was seen in the livers of the HFD + RJ-treated rats (Figure 5D). A similar picture to that seen in the HFD-treated rats was also seen in the livers of the HFD-fed rats (Figure 5E).

## 4. Discussion

The findings of this study revealed that the chronic feeding of RJ to HFD-fed rats not only reduces the gain in weight and improves IR, but also attenuates hyperglycemia and alleviates hepatic damage and steatosis. In addition, this study showed that the antidiabetic and anti-steatosis mechanisms by which RJ acts involve at least antioxidant potential, as well as the activation of the hepatic AMPK signaling-mediated upregulation of PPARα (fatty acid oxidation) and the suppression of SREBP1/2 (de novo lipogenesis).

Chronic HFD feeding is the best model to induce obesity, metabolic features, and NAFLD in rats. Obesity and associated metabolic abnormalities are the best-known risk factors for NAFLD and NASH. In addition, the higher adipose tissue mass stimulates the release of leptin from the adipose tissue, stimulating further food intake and exaggerating the increase in body weight. Indeed, the polyphagic HFD-fed animals of this study showed typical features of type 2 diabetes mellitus (T2DM), including hyperglycemia, hyperinsulinemia, impaired OGTT, IR, obesity, and dyslipidemia. These data are similar to the findings of many other studies. On the contrary, the ability of RJ to reduce body weight and reverse the other metabolic symptoms was our strongest evidence for the antidiabetic effect of RJ. These effects were expected given the previously reported studies that found the ability of RJ at doses of 100–300 mg/kg to reduce fasting glucose levels, modulate circulatory insulin levels, improve IR and HOMA-IR, lower HbA1C, and attenuate obesity in diabetic rats [25,27,32,37]. Moreover, higher doses of 1000–3000 mg/kg showed hypoglycemic effects in diabetic subjects with varied effects on insulin and HbA1C [24,26,38]. Interestingly, RJ did not alter food or calorie intake in the control or HFD-fed rats, suggesting that its hypoglycemic and hepatic protective effect is independent of modulating food/calorie intake.

Dyslipidemia remains the most common cause of the development of NAFLD [4]. In addition, oxidative stress and inflammation are the major triggers that can accelerate liver damage, hyperglycemia, and steatosis and facilitate the progression to NASH by altering enzyme activities, inducing lipid peroxidation, and promoting DNA damage and hepatic IR [39]. The relationship between oxidative stress/inflammation and IR is bidirectional. On the one hand, increased oxidative stress and inflammation in the adipose tissue induce peripheral IR [40,41]. On the other hand, the development of IR in the adipose tissue stimulates hepatic lipogenesis, oxidative stress, inflammation, and IR [39]. Within this view and in response to impaired insulin activity in the peripheral tissues, the stimulated lipolysis in the WAT enhances the influx of FFAs to the liver [39]. This stimulates de novo lipogenesis and results in the accumulation of TGs, mitochondrial damage, and the generation of ROS and inflammatory cytokines. In addition, adipose tissue IR promotes the release of numerous inflammatory cytokines that trigger mild systemic inflammation and induces hepatic inflammation and oxidative stress [4,39].

In the same line with these studies, the HFD-fed rats in this study showed increased serum and hepatic levels of FFAs, which could be attributed to the uptake, as well as increased lipolysis in the peripheral tissues of these rats due to the obvious IR. In addition, their livers showed increased fat vacuoles, which increased TGs, CHOL, IL-6, and TNF-α, concomitant with the higher serum levels of TGs, CHOL, and LDL-c. These data indicate increased lipogenesis and inflammation in the livers of these rats. This picture is similar to many previous studies that have also shown increased markers of inflammatory mediators in the livers of NAFLD animals. In addition, the livers of HFD-fed rats showed higher lipid peroxides that coincided with reduced levels of antioxidant markers (i.e., GSH and SOD), which indicates increased scavenging of these antioxidants due to high levels of ROS. Reduced levels of enzymatic and nonenzymatic antioxidants have been shown in numerous experimental animal models and in humans with NAFLD [8,42].

In contrast, treatment with both doses of RJ significantly improved the alterations in the serum and liver lipids, reduced hepatic levels of MDA, TNF-α, and IL-6, and increased hepatic levels of GSH and SOD in the HFD-fed rats. These data indicate the potent hypolipidemic and antioxidant potentials of RJ. Of note, treatment with RJ did not affect CHOL and TGs fecal levels in the control and HFD-fed rats, thus dissipating its hypolipidemic effect from altering intestinal lipid absorption. While the significant improvement could explain these data in peripheral IR and the subsequent amelioration in serum insulin levels, they may also suggest independent effects. Indeed, treatment with RJ of both doses also reduced the serum and hepatic levels of TGs and CHOL and stimulated levels of GSH and SOD, even in the control rats. However, since RJ did not affect the inflammatory markers in the control rats, it could be assumed that the anti-inflammatory effect of this nutritional food is secondary to its antioxidant effect. In addition, it could be suggested that RJ also stimulates peripheral IR by suppressing oxidative stress and inflammation.

The effects of RJ on metabolism and serum lipoprotein levels have been reported in both human and animal studies. Indeed, treatment with RJ at a dose of 350 mg/kg reduced serum CHOL and LDL-c levels in hypocholesterolemic adults [43]. It also reduced serum TGs and CHOL in a similar study in diabetic women with no change in HDL-c levels [44]. In addition, diabetic patients chronically fed RJ showed higher circulatory levels of TGs, CHOL, LDL-c, and apolipoprotein (Apo) A-I and had a reduced ratio of ApoB/Apo AI [24]. Similarly, the uptake of lyophilized RJ (333 mg/kg) attenuated the increase in serum CHOL and TGs levels in overweight nondiabetic individuals [45]. A similar reduction was found in the serum levels of CHOL, TGs, and LDL-c in diabetic rats after treatment with 100, 200, or 300 mg/kg [27,32]. Similar to our data, treatment with RJ at doses of 100–450 mg also reduced the serum and hepatic levels of TGs and CHOL in animal models of NAFLD.

In addition, the antioxidant and anti-inflammatory effects of RJ have been described in numerous experimental and clinical trials. Indeed, RJ inhibited inflammatory cytokine production in lipopolysaccharide (LPS)-treated BV-2 murine microglial cell line by suppressing NF-κB and p38/ c-Jun N-terminal kinase (JNK) signaling pathways [46]. Furthermore, reduced circulatory levels of Il-6 and c-reactive protein (CRP) were seen in asymptomatic overweight patients who had received RJ at a daily dose (333 mg/kg) [45]. RJ also reduced the bronchoalveolar lavage fluid levels of TNF-α in cancer and reduced cellular toxicity in patients treated with bleomycin [47]. It also reduced MDA levels, attenuated the reduction in glutathione peroxidase (GPx), reduced the expression of endothelial nitric oxide (eNOS) and Bax, and improved prostate histology in rodents with prostate cancer [48]. With no obvious toxicity, RJ also reduces the release of TNF-α, IL-1, and IL-6 from LPS-stimulated peritoneal macrophages in vitro [22]. RJ also reduced inflammatory cytokine production in animal models of colitis and renal inflammation [49,50]. By the same token, RJ has exceptional antioxidant activity mediated by chelating iron and scavenging superoxide, hydroxyl, and hydrogen peroxide radicals [51]. Others have also shown that RJ hydrolysate reduced the generation of ROS and stimulated the levels of SOD and GSH in LPS-treated macrophages [52]. It also inhibited ferric nitrilotriacetate (Fe-NTA)-induced lipid peroxidation in vitro [53]. In vivo, treatment with RJ attenuated cisplatin-mediated testicular damage by suppressing the generation of MDA and increasing the levels of GSH, SOD, and CAT [54]. It is also protected against carbon tetrachloride-induced hepatic damage by attenuating levels of MDA and stimulating levels of GSH and ascorbate [55]. In addition, the protective effect of RJ against cadmium-chloride-induced nephrotoxicity was associated with reduced lipid peroxidation, boosting GSH and antioxidant enzymes, and reducing the generation of TNF-α and IL-6 [56]. 

Yet, the mechanism by which RJ could exert its hypolipidemic, antioxidant, and anti-inflammatory effects is still not known. In previous research, the authors have shown the ability to increase the doses of RJ (150, 300, and 450 mg/kg) to alleviate NAFLD in rats and have attributed this to its antioxidant ability and regulation of circadian genes, including Per1 and Per 2, in the livers of ovariectomized rats [57]. In another study, the authors referred to the protective effect of RJ against NAFLD due to its antioxidant and anti-inflammatory effect, as well as regulating the metabolism of FAs such as α-linolenic acid, linoleic acid, arachidonic acid, and the biosynthesis of unsaturated fatty acids [58]. Although we have found similar antioxidant and anti-inflammatory effects, the ability of RJ to promote such beneficial effects on body weight, lipid and glucose metabolism, and liver pathology forced us to look further afield to identify other mechanisms. Therefore, we have targeted adiponectin signaling pathways based on the available data showing that adiponectin is a novel target for treating NAFLD [59].

Adiponectin is an adipokine that is released from adipose tissue. A negative relation between adiponectin levels and fat mass is reported. Levels of circulatory adiponectin are significantly reduced in obese animals and subjects, as well as in animals and patients with NAFLD. Adiponectin is an anti-inflammatory molecule and antidiabetic molecule that can improve insulin signaling and glucose and lipid metabolism by activating AMPK and suppressing the toll-like receptor-4 inflammatory pathway in the liver and muscles. Activating AMPK protected against NAFLD in several experimental studies [12,17]. In this regard, the pharmacological or genetic activation of AMPK inhibited hepatic lipogenesis by activating FA oxidation through activating PPARα [17]. It also inhibits hepatic TGs and CHOL synthesis by downregulating SREBP1 and SREBP2 and their target lipogenic genes, such as fatty acid synthase (FAS) and acetyl-CoA carboxylase (ACC-1) [17]. In addition, AMPK increases peripheral glucose uptake in the muscles by stimulating GLUT4 expression [60]. Furthermore, AMPK can inhibit cytokine production and stimulate antioxidant expression by suppressing the nuclear factor kappa beta (NF-κB) and activating the nuclear factor erythroid 2–related factor 2 (Nrf2) [61].

In this study, we have also seen a reduction in the circulatory levels of adiponectin and the reduced mRNA expression and phosphorylation of AMPKα in the livers of HFD-fed rats. In addition, these rats showed increased transcription of SREBP1, SREBP2, and FAS and a reduction in the mRNA levels of PPARα. These data support many other studies that also showed similar effects [12,17]. However, HFD did not change the total protein levels of AMPK. In the cell, AMPK is found in different forms, including AMPKα and AMPKβ. Unfortunately, we did not measure the mRNA of AMPKβ and the individual protein levels of each of these isoforms of AMPK, which may help us to explain this observation and why the livers of HFD-fed rats showed normal levels of total AMPK. In the same line with our study, several other authors have also shown reduced activities of AMPKα with no change in the total levels of AMPK [62,63]. Therefore, it could be possible that HFD exerts different effects on different isoforms of AMPK. On the other hand, treatment with RJ significantly increased the serum levels of adiponectin and concomitantly increased the hepatic phosphorylation of AMPK in HFD-fed rats. In parallel, it reduced the mRNA expression of SREBP1c, but stimulated the transcription of AMPKα and PPARα not only in the livers of HFD-treated rats but also in the livers of control rats. Interestingly, RJ did not affect circulatory levels of adiponectin or mRNA levels of AMPKα in the livers of control rats but significantly increased the rate of phosphorylation (activity) of MAPKα. This suggests that RJ stimulates the hepatic phosphorylation of AMPK without modulating the expression/levels of adiponectin, thus suggesting a novel independent mechanism of action. To support our findings, we treated the HFD-fed rats with 300 mg/kg RJ with CC, a well-known inhibitor of AMPK. In accordance, the treatment with AMPK abolished all the benefits of RJ on markers of oxidative stress, inflammation, glucose, insulin sensitivity, and lipids. Additionally, the treatment with CC did not alter the inhibitory effect of RJ on fat masses or its stimulatory effect on adiponectin in these HFD-treated rats. Therefore, these data suggest that RJ acts in an adiponectin-independent mechanism mainly through activating hepatic and possibly peripheral AMPK levels, a key mechanism that underlies its hypoglycemic, hypolipidemic, antioxidant, and anti-inflammatory effects. However, such an increase in adiponectin levels could be explained by the inhibitory effect of RJ on WAT adiposeness, which could be secondary to the improvement of insulin signaling. These data can be also supported by the study of Yoshida et al. [25], who have also shown the potential of RJ to alleviate hyperglycemia in obese diabetic KK-Ay mice through phosphorylating muscular and hepatic AMPK-mediated improvement in antioxidants and the suppression of inflammation. However, these authors have shown that treatment with RJ also increases the mRNA expression of adiponectin and its receptors.

In conclusion, this study is unique as it is the first to investigate the molecular mechanistic effect behind the hypoglycemic and anti-hyperlipidemic effects of RJ in HFD-fed rats with NAFLD. In accordance, our data support a beneficiary effect of RJ on activating hepatic AMPK. In addition, RJ seems to act by boosting enzymatic and nonenzymatic antioxidant systems in the liver. However, these data need further clinical validation.

## Figures and Tables

**Table 1:** Liver weights and levels of some markers in the serum of all experimental groups.

Parameter	Control	RJ	HFD	HFD + RJ	HFD + RJ + CC
Final liver weight (g)	4.6 ± 0.5	4.3 ± 0.42	6.7± 0.61 ^ab^	4.8 ± 0.38 ^c^	6.3 ± 0.82 ^abd^
Adiponectin (µg/mL)	39.4 ± 4.2	55.5 ± 5.7 ^a^	24.7 ± 3.8 ^ab^	39.8 ± 4.3 ^bc^	38.9 ± 4.7 ^abd^
Leptin (ng/mL)	22.4 ± 3.4	25.4 ± 4.1	56.3 ± 4.9 ^ab^	29.4 ± 3.7 ^abc^	49.3± 5.4 ^abd^
Serum TNF-α (pg/mL)	83.2 ± 7.8	91.2 ± 8.5	322 ± 19 ^ab^	125 ± 11.2 ^abc^	311± 28.2 ^abd^
Serum IL-6 (pg/mL)	46.3 ± 5.1	49.2 ± 5.3	123 ± 13.1 ^ab^	66.2 ± 6.2 ^abc^	118± 10.2 ^abd^
Serum ALT (U/L)	24.5 ± 3.8	27.3 ± 5.7	88.2 ± 5.5 ^ab^	32.3 ± 4.7 ^abc^	82.4 ± 7.5 ^abd^
AST (U/L)	35.4 ± 4.3	31.3 ± 4.9	102 ± 9.2 ^ab^	54.3 ± 5.4 ^abc^	110 ± 11.2 ^abd^
γ-GTT (U/L)	21.4 ± 4.5	25.6 ± 3.8 ^a^	69.3 ± 3.2 ^ab^	37.3 ± 4.1 ^abc^	73.2 ± 6.9 ^abd^

Data are presented as means ± SD (n = 8/group). ^a^: vs. control; ^b^: vs. RJ-treated control rats; ^c^: vs. HFD-model rats; ^d^: vs. HFD + RJ-treated rats. RJ: royal jelly; HFD: high-fat diet; CC: compound C, an AMPK inhibitor.

**Table 2:** Serum, hepatic, and fecal lipid profiles in all experimental groups.

	Parameter	Control	RJ	HFD	HFD + RJ	HFD + RJ + CC
Serum	TGs (mg/dL)	43.3 ± 5.1	34.3 ± 3.2 ^a^	89.3 ± 7.8 ^ab^	47.6 ± 5.4 ^bc^	83.4 ± 6.5 ^abd^
	CHOL (mg/dL)	84.3 ± 7.5	68.5 ± 5.3 ^a^	176 ± 13.4 ^ab^	81.1 ± 7.6 ^bc^	183 ± 15.6 ^abd^
	LDL-c (mg/dL)	43.2 ± 4.6	35.4 ± 3.5 ^a^	98.2 ± 6.5 ^ab^	54.5 ± 5.3 ^abc^	89.3 ± 7.3 ^abd^
	HDL-c (mg/dL)	22.4 ± 4.2	19.8± 3.7	12.2 ± 2.1 ^ab^	24.5 ± 0.67 ^abc^	14.3 ± 1.4 ^abd^
	FFAs (µmol/L)	882 ± 55.8	632± 43.7 ^a^	1632 ± 150.5 ^ab^	829 ± 77.2 ^bc^	1722 ± 125.2 ^abd^
Liver	TGs (mg/g)	3.6 ± 0.4	2.43 ± 0.2 ^a^	7.6 ± 0.6 ^ab^	4.3 ± 0.5 ^abc^	7.9 ± 0.6 ^abd^
	CHOL (mg/dL)	4.7 ± 0.5	3.5 ± 0.3 ^a^	8.6 ± 0.7 ^ab^	4.3 ± 0.5 ^abc^	7.9 ± 0.8 ^abd^
	FFAs (µmol/L)	245± 22.2	143± 13.2 ^a^	565 ± 43.4 ^ab^	251 ± 19.4 ^bc^	632 ± 46.7 ^abd^
Stool	TGs (mg/g dry)	2.4 ± 0.35	2.6 ± 0.27	6.34 ± 0.73 ^ab^	5.9 ± 0.53 ^ab^	6.7 ± 0.74 ^ab^
	CHOL (mg/g dry)	4.32 ± 0.53	4.02 ± 0.38	8.34 ± 0.82 ^ab^	7.7 ± 0.93 ^ab^	8.3 ± 0.7 ^ab^

Data are presented as means ± SD (n = 8/group). ^a^: vs. control; ^b^: vs. RJ-treated control rats; ^c^: vs. HFD-model rats; ^d^: vs. HFD + RJ-treated rats. RJ: royal jelly; HFD: high-fat diet; CC: compound C, an AMPK inhibitor.

## Data Availability

The datasets used and analyzed in the current study are available from the corresponding author upon reasonable request.
